# Substrate Deprivation Therapy to Reduce Glycosaminoglycan Synthesis Improves Aspects of Neurological and Skeletal Pathology in MPS I Mice

**DOI:** 10.3390/diseases5010005

**Published:** 2017-02-23

**Authors:** Ainslie L. K. Derrick-Roberts, Matilda R. Jackson, Carmen E. Pyragius, Sharon Byers

**Affiliations:** 1 Genetics and Molecular Pathology, SA Pathology, North Adelaide, SA 5006, Australia; matilda.jackson@adelaide.edu.au (M.R.J.); carmen.macsai@y7mail.com (C.E.P.); sharon.byers@adelaide.edu.au (S.B.); 2 Paediatrics and Reproductive Health, The University of Adelaide, Adelaide, SA 5005, Australia; 3 School of Molecular & Biomedical Science, The University of Adelaide, Adelaide, SA 5005, Australia

**Keywords:** mucopolysaccharidosis type I, substrate deprivation, rhodamine B, lysosomal storage disorder, glycosaminoglycans

## Abstract

Mucopolysaccharidosis type I (MPS I) is the most common form of the MPS group of genetic diseases. MPS I results from a deficiency in the lysosomal enzyme α-l-iduronidase, leading to accumulation of undegraded heparan and dermatan sulphate glycosaminoglycan (GAG) chains in patient cells. MPS children suffer from multiple organ failure and die in their teens to early twenties. In particular, MPS I children also suffer from profound mental retardation and skeletal disease that restricts growth and movement. Neither brain nor skeletal disease is adequately treated by current therapy approaches. To overcome these barriers to effective therapy we have developed and tested a treatment called substrate deprivation therapy (SDT). MPS I knockout mice were treated with weekly intravenous injections of 1 mg/kg rhodamine B for six months to assess the efficacy of SDT. Mice were assessed using biochemistry, micro-CT and a battery of behaviour tests to determine the outcome of treatment. A reduction in female bodyweight gain was observed with the treatment as well as a decrease in lung GAG. Behavioural studies showed slight improvements in inverted grid and significant improvements in learning ability for female MPS I mice treated with rhodamine B. Skeletal disease also improved with a reduction in bone mineral volume observed. Overall, rhodamine B is safe to administer to MPS I knockout mice where it had an effect on improving aspects of neurological and skeletal disease symptoms and may therefore provide a potential therapy or adjunct therapy for MPS I patients.

## 1. Introduction

The mucopolysaccharidosis (MPS) disorders are a group of inherited metabolic diseases resulting from abnormalities in enzymes required for the turnover of intracellular glycosaminoglycan (GAG) chains. MPS I is considered the classic MPS type and is the most common MPS occurring in approximately 1 in 100,000 newborns worldwide; however incidence and prevalence of phenotypic groups vary from region to region [1,2]. It is caused by a reduction in, or a deficiency of, the lysosomal enzyme α-l-iduronidase (IDUA, E.C. 3.2.1.76), which is required for the intracellular degradation of heparan sulphate (HS) and dermatan sulphate (DS) GAG chains. In the absence of IDUA, HS and DS GAGs progressively build up within cells, disrupting normal cell function and resulting in multiple organ failure. Both severe and attenuated forms of MPS I are observed, reflecting differences in residual enzyme activity [3]. Because of the widespread build-up of HS and DS GAGs, affected children display a range of symptoms including organomegaly, skeletal abnormalities, mental deterioration and corneal clouding. Death most commonly arises from cardiac or respiratory failure in early adolescence.

Because of the range of organs affected by MPS I and the progressive nature of pathology, finding an effective multi-tissue treatment for MPS I has been difficult, as indeed it has been for all MPS types. Bone marrow transplantation (BMT) and enzyme replacement therapy (ERT) are currently in the clinic; however, each has its own limitations.

Bone marrow transplantation has been shown to be effective in prolonging life and reducing some clinical symptoms associated with MPS I, such as slowing down central nervous system (CNS) deterioration [4,5]. However, this improvement is generally only seen when transplantation is undertaken at a very early age (<2 years with 6–12 months being the optimal age) and therefore before the onset of significant CNS pathology. Bone pathology also responds to BMT but the outcome is variable [6] and orthopaedic procedures are still needed after successful BMT. Other problems associated with BMT include the high mortality rate (10%–20% in the first year post-BMT) and finding a suitable donor before the onset of symptoms [7]. Despite these limitations, BMT remains the treatment of choice for MPS I patients with CNS involvement who are 2 years old or less.

ERT using recombinant IDUA (Aldurazyme^®^, Genzyme, Cambridge, MA, USA) was introduced in 2003 (USA). Advantages of ERT over BMT are the higher level of circulating enzyme that can be achieved and the negation of graft-versus-host disease. Patients on ERT have improved heart and lung function, reduced urinary GAG excretion and decreased liver and spleen size [8]. Skeletal disease response to ERT treatment is variable, with limited improvement seen in some cases [9]. Joint flexion shows a small improvement after ERT but improvement soon plateaus [9] and joint function does not normalise [9]. Corneal clouding also does not respond to ERT [9]. With a molecular weight of 87 kDA [10]**,** recombinant α-l-iduronidase is too large to cross the blood-brain barrier and as such is ineffective in treating the neurological aspect of the disease. MPS I patients with CNS disease are therefore not placed on ERT regimens. However, there is a role for ERT in stabilising respiratory disease in MPS I patients with CNS disease prior to BMT [11,12].

The limitations of current therapies, especially towards the major pathologies observed in MPS I (brain and skeletal disease), have led to an intense research effort by many laboratories to devise alternative approaches that can be used as either stand-alone therapies or adjuvant therapies to ERT or BMT. Substrate deprivation or reduction therapy (SDT/SRT) reduces the initial synthesis of the GAG chains that are the substrate for the MPS enzyme and restores the balance between synthesis and degradation of GAG in MPS cells. The advantage of SDT over ERT/BMT lies in the use of small molecular weight chemical inhibitors of GAG synthesis that are capable of crossing the blood-brain barrier or diffusing into poorly vascularised tissues such as cartilage and cornea.

The isoflavone genistein has been used as an SDT/SRT candidate for reducing GAG synthesis. Studies in MPS II mice have shown decreased urinary, liver, spleen and heart GAG, as well as a small reduction in brain GAG storage at low concentrations, with a higher dose of genistein not providing any additional benefit [13]. MPS IIIB mice treated with genistein showed reduced tissue GAG content [14] and a correction in aberrant open field exploration [15].

These promising results in mice led to human clinical trials to treat MPS IIIA and MPS IIIB patients, which showed decreased urinary GAG, improved hair composition, improved/stabilised sleep habits, improved general behaviour, speech performance and comprehension in some patients as well as improved or stabilised disease in some patients [16,17]. No long-term reports (>3 years) of patient use have been reported for genistein. A double-blinded, placebo-controlled 6-month clinical trial showed no statistically significant clinical improvement in treated patients, despite significantly lower levels of urinary GAG detected in treated patients [18] and higher doses (15 mg/kg/day) did not correlate to significantly improved behaviour despite a dose-dependent decrease in urinary GAG observed [19]. A supra-high dose genistein (150 mg/kg/day) trial in MPS patients with CNS disease showed limited effect on disease, potential estrogenic side effects and variable effect on urinary GAG excretion [20]. Studies have also shown limited improvement in shoulder joint range of motion in MPS II patients [21]). No adverse reactions were observed in any of these studies to genistein treatment.

Our laboratory has developed and tested substrate deprivation therapy (SDT) using rhodamine B (also termed substrate reduction therapy) for MPS disorders [22,23,24]. While we have shown that this approach using rhodamine B improved somatic [24] and neurological function in the MPS IIIA mouse [23], its effect on the multiple sites of pathology in MPS I is unknown. MPS I presents with subtle differences in CNS disease progression compared to MPS III disease, which can present with severe aggression and temperament issues not generally seen in MPS I patients [2,25,26,27]. MPS I pathology also has a skeletal disease component not seen in MPS IIIA, which may affect the clinical efficacy. Before we can put forward SDT as a therapy for the pathology observed in MPS I, it must be trialled in the appropriate animal model.

There are animal models of MPS I in the cat [28], dog [29] and mouse [30], with the latter being well characterised. The MPS I knockout mouse (*Idua*^−/−^) [30] displays somatic pathology that includes increased urinary output, widespread lysosomal storage as determined by histology and severe skeletal and physical abnormalities [30,31]. Behaviour deficits are also observed as measured by open field exploration, marble burying anxiety, spatial learning and memory and novel object recognition [32]. This mouse model is an accurate model of human MPS I disease and is a valuable tool for characterising disease progression and the evaluation of new therapies.

In this study, we report SDT treatment of MPS I mice using rhodamine B, a non-specific inhibitor of GAG synthesis. In a six-month *in vivo* therapy trial, rhodamine B altered several clinical parameters of disease progression toward normal including a reduction in female bodyweight gain, decreased lung GAG, decreased bone mineral volume and treated female mice showed improved learning ability as assessed by the water cross maze. This suggests that substrate deprivation therapy should not be overlooked in models without residual enzyme activity particularly as an adjunct therapy.

## 2. Materials and Methods

### 2.1. Animal Maintenance

Mice were obtained from the Jackson laboratory on a C57Bl/6 background and maintained in-house. Genotype was determined in the first week after birth by PCR of genomic DNA as previously described [30] and pups were weaned from the dam at 3 weeks of age. Mice were housed in a 14 h/10 h light/dark cycle with *ad libitum* access to food and water. Normal, MPS I untreated and MPS I mice treated with Rhodamine B were weighed weekly to record bodyweight measurements as an overall measure of health using normal (*n* = 9–15 female, *n* = 10–14 male), MPS I untreated (*n* = 6–14 female, *n* = 6–17 male) and MPS I SDT (*n* = 8 female and *n* = 8 male) mice. Treatment was carried out with intravenous weekly injections of 1 mg/kg rhodamine from 4 weeks of age, as previously described [23,24]. Mice were sacrificed by carbon dioxide asphyxiation and tissues collected for subsequent analysis. All animal studies were approved by the Children, Youth and Women’s Health Service and The University of Adelaide Animal Ethics Committees. Animal numbers used for each experiment can be found in Supplementary Table S1.

### 2.2. Behaviour Testing

All behaviour testing was conducted between 0800h and 1100h under normal lighting conditions by the same experimenter (M.R.J.). Testing was carried out at monthly intervals from 1 month of age for open field, rotarod and inverted grid. Open field testing was carried out using an Animal Activity Monitoring System (Harvard Apparatus, Holliston, MA, USA) as previously described [33,34] on normal (*n* = 9 female, *n* = 10 male), MPS I untreated (*n* = 10 female, *n* = 8 male) and MPS I SDT (*n* = 8 female, *n* = 8 male) mice. Motor coordination and balance was assessed using the Rota-Rod system with CUB 2005 data acquisition software (Ugo Basile Biological Research Apparatus) as previously described [33,34] using the same mice as in the open field test. Briefly, mice were placed on a rod rotating at 5 rpm and the speed increased to 35 rpm over 120 s. A constant speed of 35 rpm was continued for a further minute, with a maximum time of 180 s. If mice failed to remain on the rod or went one full revolution around the rod, they were manually stopped and the time and speed recorded. Neuromuscular strength was assessed using the inverted grid test [33,34] using normal (*n* = 11 female, *n* = 10 male), MPS I untreated (*n* = 11 female, *n* = 10 male) and MPS I SDT (*n* = 8 female, *n* = 8 male) mice. 

Water cross maze testing was carried out as previously described [23,33,34]. Firstly, naïve mice were tested in the learning phase at 4 months of age with long-term monthly memory sessions up to 6 months of age in normal (*n* = 3 female, *n* = 5 male), MPS I untreated (*n* = 6 female, *n* = 4 male) and MPS I SDT (*n* = 6 female, *n* = 4 male) mice. Parameters recorded were escape latency (number of seconds to find submerged platform, to a maximum of 60 s), incorrect entries/re-entries (entry or re-entry into an incorrect arm) and correct entries (mouse goes directly to platform from release point). Naïve mice were also assessed for learning capacity at 6 months of age for normal (*n* = 4 female, *n* = 4 male), MPS I untreated (*n* = 4 female, *n* = 4 male) and MPS I SDT (*n* = 2 female, *n* = 4 male) mice.

### 2.3. Determination of Tissue GAG

Post-mortem tissue samples from normal (*n* = 5 female, *n* = 7 male), MPS I (*n* = 7 female, *n* = 3 male) and MPS I SDT (*n* = 6 female, *n* = 7 male) mice were stored at −20 °C until homogenised in 0.1% (*w*/*v*) Triton X-100 using manual Teflon to glass tissue homogenisers followed by centrifugation at 10,000× *g* for 15 min to isolate tissue supernatant. Aliquots of each supernatant were precipitated with cetylpyridium chloride citrate as previously described [23,33,34]. GAG was determined as uronic acid [35] and normalised to wet tissue weight (grams).

### 2.4. Determination of Lysosomal Enzyme Activity

Aliquots of triton extracted tissue supernatants were used to determine lysosomal enzyme activity. β-hexosaminidase and α-l-iduronidase activity were determined as previously described [30,36] and normalised to wet tissue weight (grams). Results are expressed as nmol/min/mg tissue.

### 2.5. Extraction and Quantification of Brain Monosialogangliosides

Mouse brains were cut in half longitudinally, along the cerebral fissure. Gangliosides were isolated using a modified Folch method combined with solid phase extraction according to Williams et al [37,38] as previously described [33,39] and quantified by electrospray ionization tandem mass spectrometry. G_M1_ (d18:1/18:0), G_M2_ (d18:1/22:1), G_M3_ (d18:1, 18:0), and *d3*-G_M1_ (d18:1, 18:0) were quantified against a *d3*-G_M1_ internal standard and normalised to tissue weight as previously described [33,39]. Results are calculated as nmol/g wet tissue weight and expressed as a percentage of untreated normal values.

### 2.6. Ex Vivo Micro-CT

*Ex vivo* micro-CT of the L5 vertebrae was performed as previously described [33,34,40]. Height and width measurements, and quantitative evaluation of bone structure, including bone mineral volume, trabecular number, trabecular thickness and bone surface density was determined using CTAn (v1.9.3.2., Bruker microCT (formerly SkyScan), Kontich, Belgium). Analysis was conducted on normal (*n* = 3 female, *n* = 3 male), MPS I untreated (*n* = 6 female, *n* = 3 male) and MPS I SDT (*n* = 3 female, *n* = 3 male) mice.

### 2.7. Statistics

All statistical analysis was conducted using either one-way or two-way analysis of variance (ANOVA) where appropriate, with a Tukey’s post-hoc test used to measure statistical significance between designated variables (SigmaStat v3.0).

## 3. Results

### 3.1. Bodyweight Profiles

**Figure 1:**
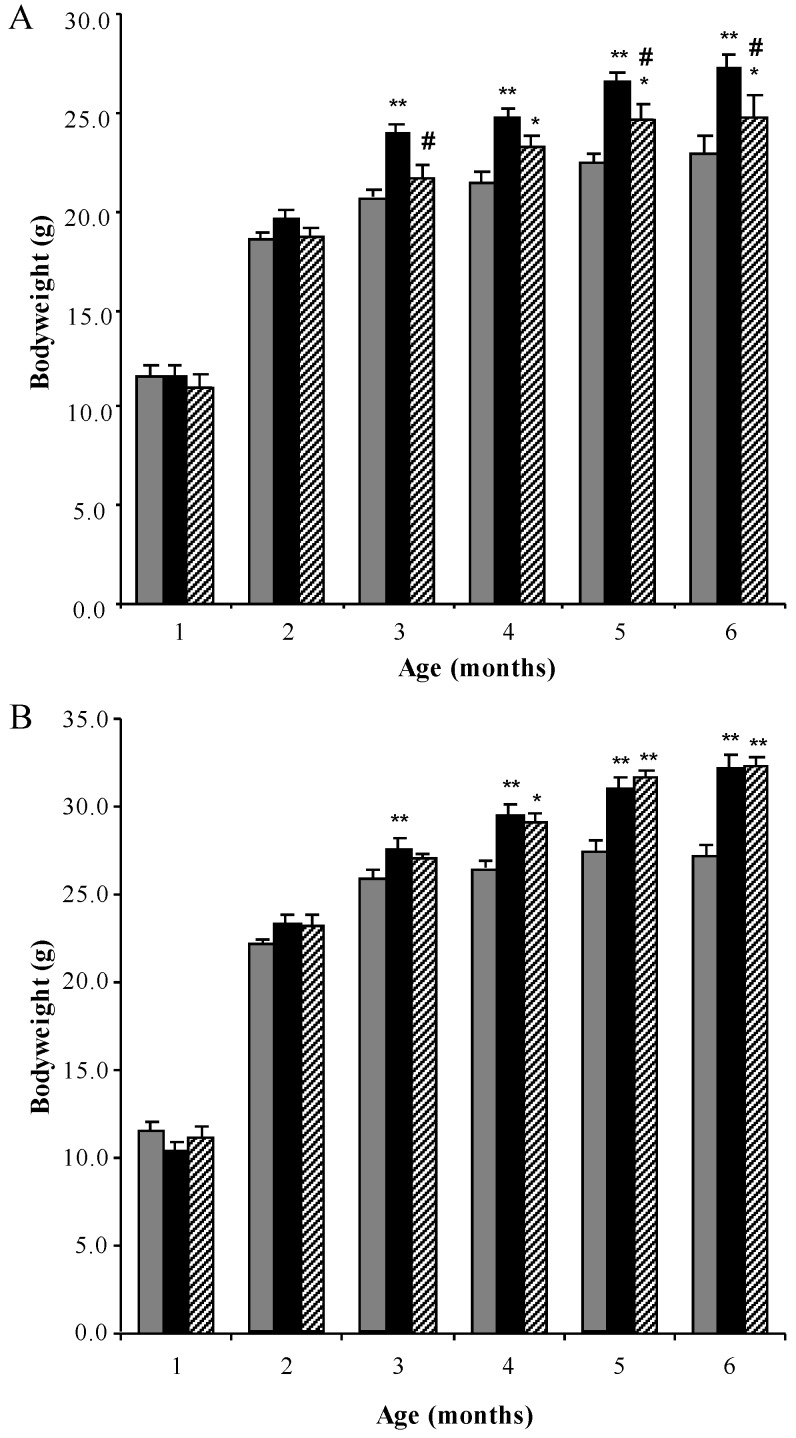
Bodyweight analysis. Monthly bodyweights (grams) of normal (grey bars), MPS I untreated mice (black solid bars) and MPS I mice treated with rhodamine B (black hatched bars) in female (**A**) and male (**B**) mice respectively. Data is presented as the average + SEM for each group. * and ** indicates significantly different from normal (*p* < 0.05 and *p* < 0.001 respectively, two-way ANOVA, Tukey’s post-hoc test). ^#^ indicates MPS I treated significantly different from MPS I untreated (*p* < 0.05, two-way ANOVA, Tukey’s post-hoc test).

MPS I mice were treated from 4 weeks of age with weekly intravenous injections of 1 mg/kg rhodamine B until 6 months of age. Bodyweights of male and female mice were measured weekly. All MPS I untreated mice gained weight more rapidly than normal control mice, with MPS I untreated females and males being significantly different from 3 months of age (Figure 1A and B respectively, *p* < 0.001). A decrease in bodyweight gain was observed in MPS I female mice treated with rhodamine B, which was significantly less than MPS I untreated mice at 3, 5 and 6 months of age (Figure 1A). A small delay in bodyweight gain was observed in male MPS I mice treated with rhodamine B, with treated males not becoming statistically different to normal male mice until 4 months of age.

### 3.2. Biochemical Analysis

#### 3.2.1. Effect of SDT Treatment on Primary and Secondary Storage

**Figure 2:**
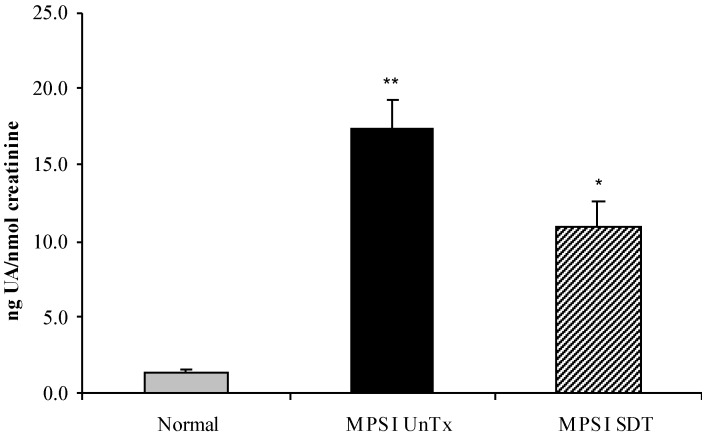
Urinary GAG levels. Normal (grey bars), MPS I untreated mice (black solid bars) and MPS I mice treated with rhodamine B (black hatched bars) urinary GAG was analysed at 6 months to determine GAG levels. Aliquots of urine were cpc/citrate precipitated and uronic acid was determined. Uronic acid is expressed as ng total uronic acid and normalised to nmoles of creatinine. Data is expressed as average + SEM for each group. * and ** indicates significantly different from normal (*p* < 0.05 and *p* < 0.001 respectively, one-way ANOVA, Tukey’s post-hoc test).

**Figure 3:**
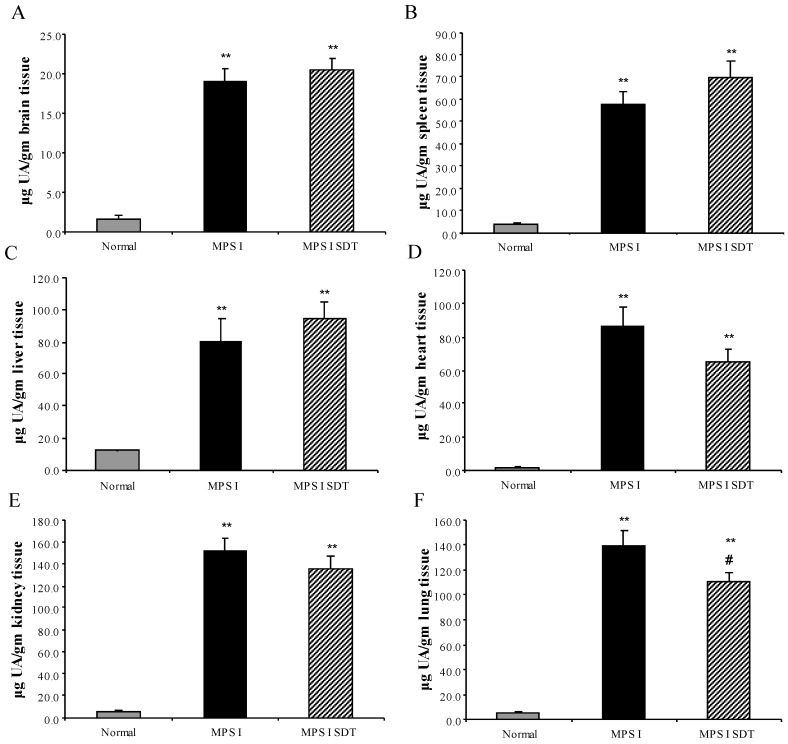
Tissue GAG levels. Normal (grey bars), MPS I untreated mice (black solid bars) and MPS I mice treated with rhodamine B (black hatched bars) were analysed post-mortem at 6 months to determine GAG levels. Aliquots of triton extracted tissues were cpc/citrate precipitated and uronic acid was determined. Uronic acid is expressed as µg total uronic acid for brain (**A**), spleen (**B**), liver (**C**), heart (**D**), kidney (**E**) and lung (**F**). Data is expressed as average + SEM for each group. ** indicates significantly different from normal (*p* < 0.001, one-way ANOVA, Tukey’s post-hoc test). ^#^ indicates MPS I treated significantly different from MPS I untreated (*p* < 0.001, one-way ANOVA, Tukey’s post-hoc test).

No difference between sexes was observed for tissue GAG or ganglioside content and both sexes were combined for analysis. Urinary GAG was analysed at 6 months of age (Figure 2), with MPS I untreated showing 12.8-fold higher excretion of GAG than age-matched normal mice (*p* < 0.001). MPS I SDT mice only had an 8-fold increase compared to normal mice, however, this was not statistically significant (*p* > 0.05) to untreated MPS I mice (Figure 2). Tissue GAG was elevated in all tissues analysed (liver, kidney, spleen, heart, lung and brain) in MPS I untreated mice compared to age-matched normal controls (Figure 3, *p* < 0.001). Treatment of MPS I mice with rhodamine B did not alter tissue GAG levels in liver, kidney, heart, spleen and brain (Figure 3, *p* > 0.05). The exception to this was lung GAG, which was significantly decreased in MPS I mice treated with rhodamine B (Figure 3F, *p* < 0.05), however still remained over 20-fold higher than normal indicating a modest improvement. Secondary storage of brain G_M2_ and G_M3_ gangliosides was observed at 6 months with a 3.3- and 3-fold increase in G_M2_ and G_M3_ ganglioside respectively, which was significantly elevated in MPS I untreated mice compared to the age-matched normal control (Table 1, *p* < 0.001). No effect on G_M2_ and G_M3_ levels was observed with rhodamine B treatment in MPS I mice. Urinary GAG was elevated in MPS I untreated mice, and was reduced in MPS I SDT mice compared to untreated MPS I mice, but this did not reach significance (*p* > 0.05).

#### 3.2.2. Tissue Enzyme Levels

No difference was observed between the sexes for enzyme activities and data from both sexes were combined for analysis. β-hexosaminidase and α-l-iduronidase levels (data not shown) were determined for liver, kidney, spleen, heart, lung and brain (Table 2). Compared to normal, MPS I mouse tissues showed significant elevations in β-hexosaminidase activity and, as expected, significant decreases in α-l-iduronidase (data not shown). No change in β-hexosaminidase compared to MPS I untreated mice was observed with treatment (*p* > 0.05).

### 3.3. Effect of SDT Treatment on Behaviour

#### 3.3.1. Inverted Grid

**Figure 4:**
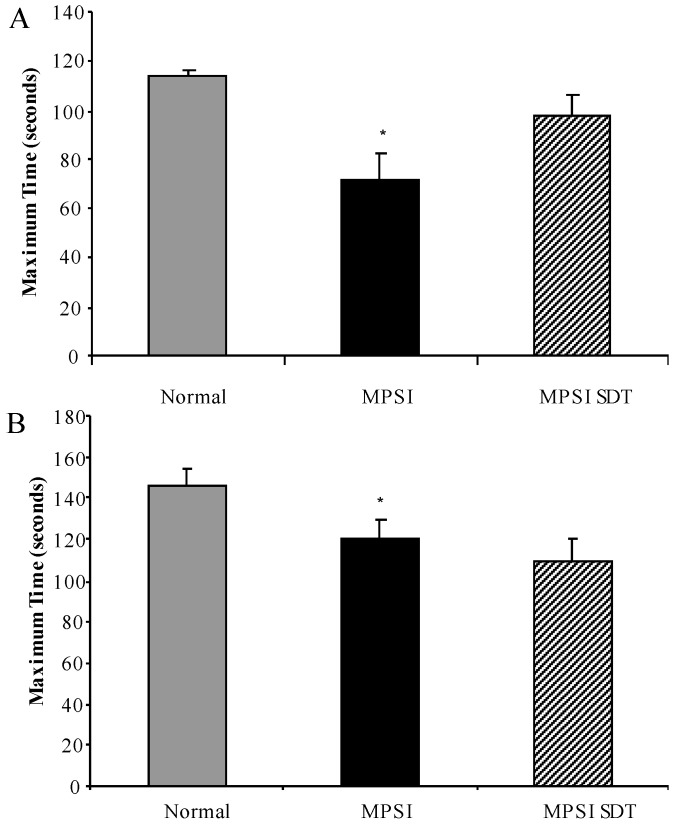

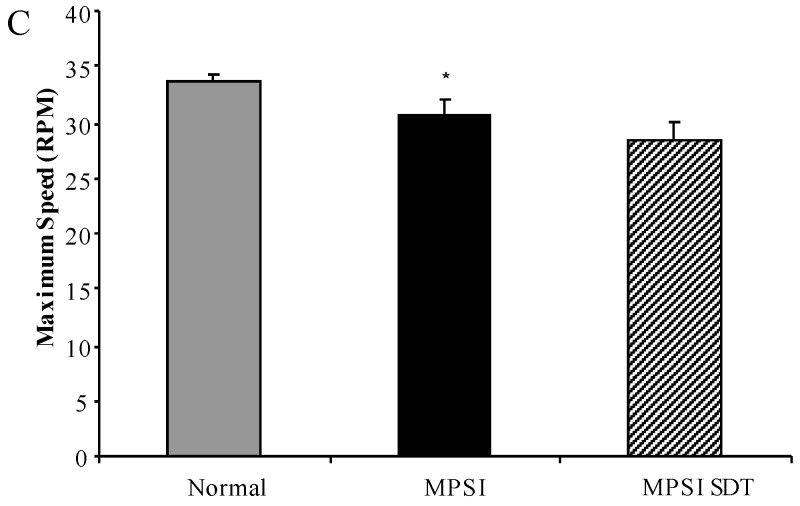
Motor function testing. Normal (grey bars), MPS I untreated mice (black solid bars) and MPS I mice treated with rhodamine B (black hatched bars) were tested in motor function tests at 6 months of age. Inverted grid latency (**A**) was measured over a period of 2 min at 6 months of age. Mice were tested in the rotarod which measured maximum time (**B**) and maximum speed (**C**) reached. Data is expressed as average + SEM for each group. * indicates significantly different from normal (*p* < 0.05, one-way ANOVA, Tukey’s post-hoc test).

No sex difference was observed on the inverted grid and data from both sexes was combined. MPS I untreated mice had a reduction in time spent on the inverted grid compared to normal mice at 6 months of age (Figure 4A, *p* < 0.05), MPS I treated mice had improved latency compared to MPS I untreated mice, however this did not reach statistical significance (Figure 4A, *p* > 0.05).

#### 3.3.2. Rotarod

No sex difference was observed on the rotarod and data from both sexes was combined. MPS I untreated mice had a reduction in maximum time achieved on the rotarod, which was significantly different to normal at 6 months of age (Figure 4B, *p* < 0.05). As well as a reduction in time, MPS I mice had a slight reduction in maximum speed achieved on the rotarod, which reached significance compared to normal at 6 months of age (Figure 4C, *p* < 0.05). No significant differences were noted between MPS I untreated mice and MPS I mice treated with rhodamine B (*p* > 0.05) in either time or speed.

#### 3.3.3. Open Field Exploration

**Figure 5:**
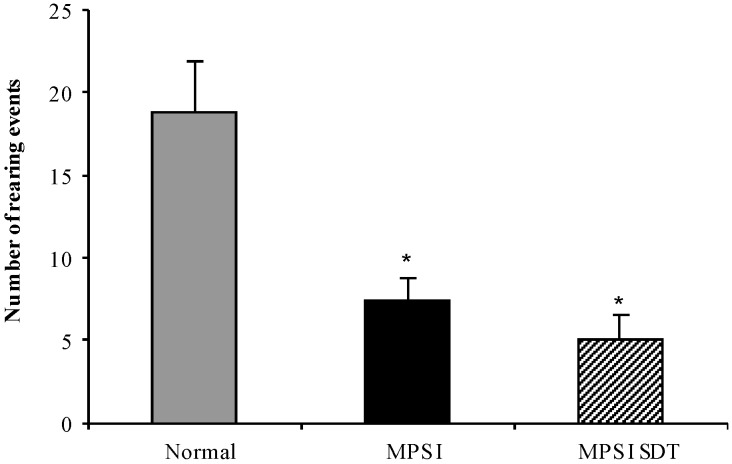
Open field exploration. Normal (grey bars), MPS I untreated mice (black solid bars) and MPS I mice treated with rhodamine B (black hatched bars) were tested in the open field apparatus over a period of 3 min at 6 months of age. Rearing events were recorded and data is expressed as average + SEM for each group. * indicates significantly different from normal (*p* < 0.05, one-way ANOVA, Tukey’s post-hoc test).

No sex difference was observed in the open field and data from both sexes was combined. Rearing activity was significantly reduced in MPS I untreated mice compared to normal mice at 6 months of age (Figure 5, *p* < 0.05). Treatment with rhodamine B had no effect on rearing (*p* > 0.05). No significant differences in activity as determined by marginal or centre distance (% total distance) were observed in any group (data not shown).

#### 3.3.4. Water Cross Maze

**Figure 6:**
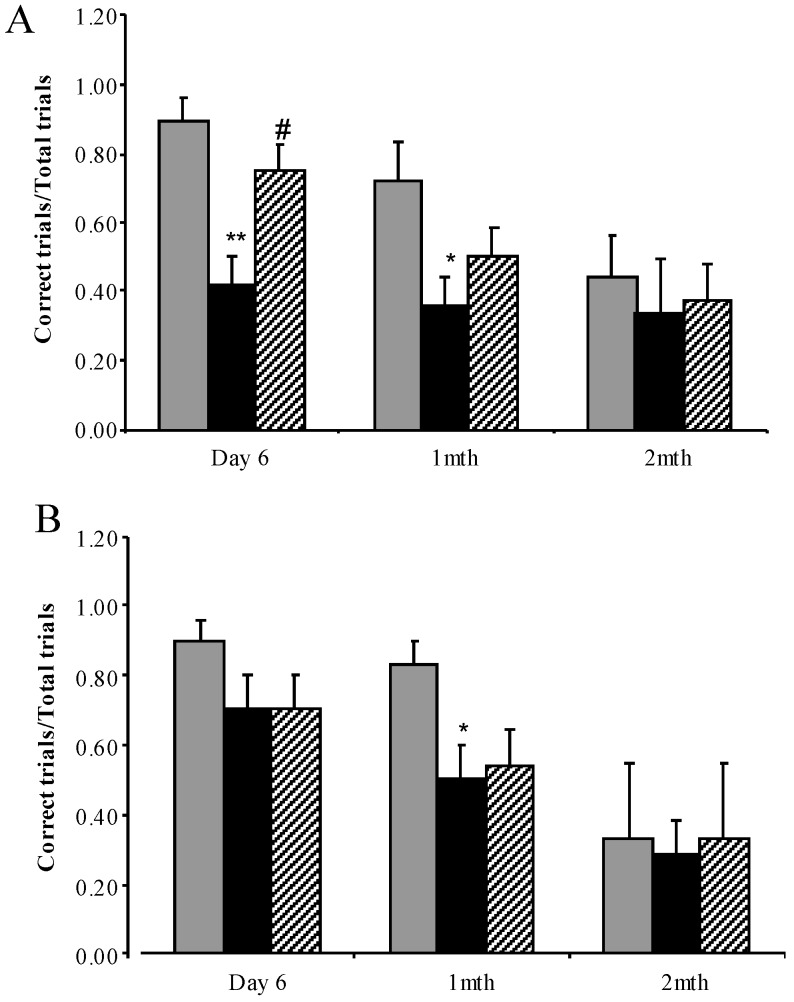
Water cross maze testing to assess spatial learning and memory. Sex separated correct entries analysis assessed in the water cross maze at 4 months of age in normal mice (grey bars), MPS I untreated mice (black solid bars) and MPS I mice treated with rhodamine B (black hatched bars). Correct entry learning and memory data combined is presented for female (**A**) and male (**B**) mice. Results are expressed as average + SEM of six trials for each mouse tested. * and ** indicate significantly different from normal (*p* < 0.05 and *p* < 0.001 respectively, one-way ANOVA, Tukey’s post-hoc test). ^#^ indicates significant difference in MPS I treated versus MPS I untreated mice (*p* < 0.05, one-way ANOVA, Tukey’s post-hoc test).

A difference was observed between male and female performance in the water cross maze learning component undertaken at 4 months of age. While both female and male mice learnt the spatial learning task, untreated female MPS I mice had a significantly reduced ability to locate the platform (41.7% correct trials compared to 88.9% correct trials in normal mice, Figure 6A). Treatment with rhodamine B resulted in a significant increase in correct entries by female mice (75%). In contrast, although the percentage of correct entries was reduced in male untreated MPS I mice (70.8% vs. 90% in normal mice) this reduction was not significant and no improvement was observed after rhodamine B administration (70.8% in treated male mice, Figure 6B).

After the learning phase, mice were re-tested 1 and 2 months later to determine the effect of treatment on memory. Both female (Figure 6A) and male (Figure 6B) untreated MPS I mice showed decreased number of correct entries compared to normal mice 1 month post-learning and all groups of mice were similar 2 months after learning. Treatment did not improve memory with no difference observed between the untreated and the treated MPS I groups for either sex (*p* > 0.05).

### 3.4. Effect of SDT Treatment on Skeletal Disease

**Figure 7:**
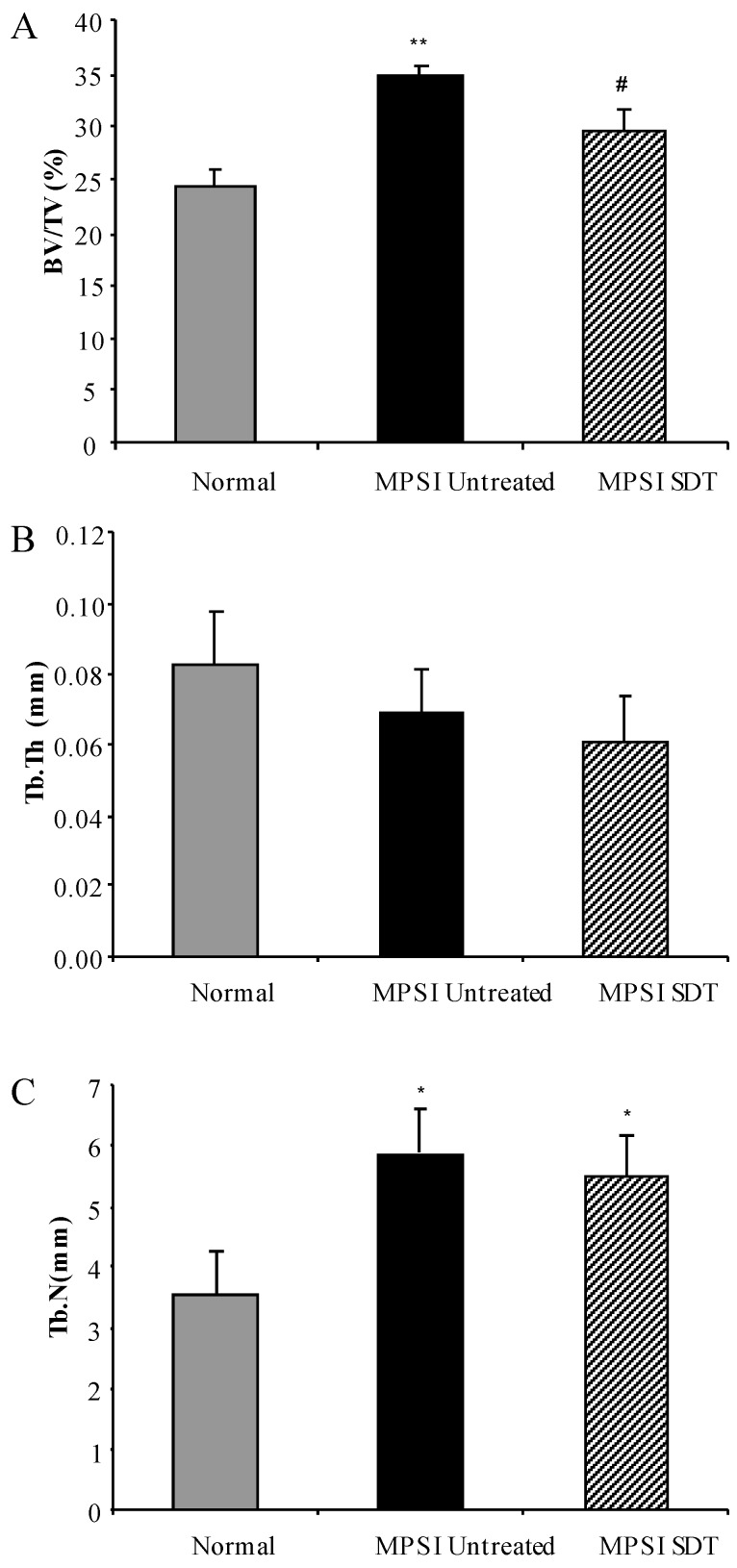
Ex Vivo micro-CT of L5 vertebrae. Normal (grey bars), MPS I untreated mice (black solid bars) and MPS I mice treated with rhodamine B (black hatched bars) were analysed using ex vivo micro-CT at 6 months. 3D analysis of reconstructed slices yielded quantitative measurements of bone mineral volume (**A**), trabecular thickness (**B**) and trabecular number (**C**). Data is presented as average + SEM. * and ** indicate significantly different from normal (*p* < 0.05 and *p* < 0.001 respectively, one-way ANOVA, Tukey’s post-hoc test, # denotes MPS I treated significantly different from MPS I untreated (*p* < 0.05, one-way ANOVA, Tukey’s post hoc test).

No sex difference was observed in the micro-CT analysis and data from both sexes was combined. Reconstructed images of L5 vertebrae demonstrated an increase in trabecular bone in untreated MPS I mice compared to normal, which decreased upon treatment. Quantitative 3D analysis confirmed a significant increase in bone mineral volume (BV/TV) in untreated MPS I mice compared to normal (34.95% ± 0.92% vs. 24.32% ± 1.43%, respectively) (Figure 7A, *p* < 0.001). Treatment of MPS I mice with rhodamine B significantly decreased BV/TV to 29.62% ± 1.92% (Figure 7A, *p* < 0.05). Trabecular thickness (Tb.Th.) was reduced in MPS I mice compared to normal, but this was not significant (Figure 7A, *p* > 0.05). MPS I mice had significantly more trabeculae (Tb.N.) than normal mice (5.89 ± 0.70 per mm in untreated vs. 3.53 ± 0.72 per mm in normal) and this was unchanged with treatment (*p* > 0.05) (Figure 7C). No alteration in vertebral height or vertebral width (data not shown) was observed with treatment compared to MPS I untreated mice.

## 4. Discussion

SDT targeting inhibition of GAG synthesis is an emerging therapy for MPS disorders. MPS I is the most common MPS disease with storage of dermatan (DS) and heparan sulphate (HS) GAGs resulting in neurological and skeletal pathology. Because of the range of organs affected by MPS I and the progressive nature of pathology, finding an effective multi-tissue treatment for MPS I has been difficult, as indeed it has been for all MPS types. SDT has the advantage of using small molecules which can diffuse into typically hard-to-reach tissues. Genistein has been used in MPS animal models, which showed an improvement in tissue GAG [13,14] and also improved behaviour in MPS IIIB [15]. Clinical trials with genistein have shown variable effect on MPS disease with slight improvement in cognition or stabilisation of disease in some patients, [16,17,18] with higher doses not correlating to improved behaviour outcome [19,20].

Rhodamine B is a non-specific inhibitor of GAG synthesis and has been used *in vitro* and *in vivo* in MPS IIIA, where an improvement in somatic and neurological pathology was observed [23,24]. The aim of this study was to determine if SDT alters CNS and skeletal pathology in the knockout MPS I mouse model when residual enzyme activity is minimal or absent. Although no adverse effects have been observed in mouse studies [22,23,24], reports of rhodamine B use in humans are limited. One study reports that an acute overexposure to aerosolised rhodamine B in humans resulted in transient mucous membrane and skin irritation, however, these symptoms resolved within 24 h [41], while acute overexposure to oral rhodamine B resulted in increased urinary excretion of the compound but no other symptoms [42]. Studies in MPS IIIA mice show no effect on litter size or liver function, with low levels of rhodamine B safe to administer in mice *in* *utero* and over multiple generations [22].

MPS I mice treated with weekly injections of 1 mg/kg rhodamine B showed no side-effects and female bodyweights were significantly reduced compared to their untreated MPS I littermates. A slight delay in male bodyweight gain was observed with treatment however, overall, treatment had little effect on male weight gain. This is in contrast to previous studies in our laboratory in MPS IIIA mice which showed a reduction in bodyweight gain in both males and females [24], suggesting that the mechanism of weight gain may be different in MPS I compared to MPS IIIA.

Tissue GAG was elevated in MPS I untreated mice in all tissues examined compared to normal mice. Treatment with rhodamine B resulted in a significant reduction in the level of GAG in the lung, with no change in tissue GAG levels in liver, kidney, spleen, heart and brain. Again, this is in contrast to our results in the MPS IIIA mouse in which tissue GAG decreased in rhodamine B treated mice [23,24]. The MPS I mouse model is a knockout with no detectable residual enzyme activity, whereas the MPS IIIA mouse model retains 3%–4% residual enzyme activity [30,43]. Because tissue GAG levels reflect the rate of synthesis (reduced with rhodamine B) combined with the rate of degradation (nil in MPS I, reduced but still present in MPS IIIA), it is possible that by 6 months of age the cumulative amount of GAG storage in treated MPS I mice reached the same level as untreated MPS I mice. This notion can be tested by short-term rhodamine B treatment of MPS I mice to determine if there is a transient decrease or delay in accumulation of GAG.

MPS I untreated mice also showed significantly elevated levels of β-hexosaminidase and significantly reduced α-l-iduronidase enzyme activity. No change in either enzyme activity was observed in MPS I mice treated with rhodamine B, suggesting that rhodamine B’s mode of action is specifically targeted to inhibition of GAG synthesis and not stabilisation of enzymes [44].

MPS I mice display behavioural deficits in the water cross maze, open field, rotarod and inverted grid tests reflecting their CNS and skeletal pathology [32,45] (Figure 4, Figure 5 and Figure 6). SDT with rhodamine B improved learning capacity in female mice but was unable to preserve long-term memory. Although male MPS I mice had a reduced learning capacity this was not significantly different to normal mice, and treatment had no effect. The reason for this difference in behaviour between the sexes, which both accumulate similar levels of brain GAG and ganglioside, is unknown. We have previously shown that both male and female MPS IIIA mice display learning deficits in the water cross maze [23], again indicating differences in the manifestation of CNS pathology in MPS I and MPS IIIA. SDT also improved performance on the inverted grid test, which may reflect an improvement in skeletal disease. SDT did not alter anxiety or balance/co-ordination as indicated by open field and rotarod tests respectively. In view of the differences observed between the response of MPS I and MPS IIIA mice to SDT in biochemical and behavioural tests, a study of MPS IIIA response to SDT using the open field and rotarod tests is currently underway.

MPS I also develop skeletal pathology as shown by a significant increase in L5 vertebral bone mineral volume, primarily due to an increase in trabecular number. BV/TV was significantly decreased in rhodamine B treated MPS I mice. This is the first report of SDT targeting inhibition of GAG synthesis to have a positive outcome on bone. Skeletal disease is not adequately addressed using current therapies and this could provide a potential treatment for reaching this disease site. Improvement in the inverted grid may also be related to improvement in skeletal disease.

In agreement with previous data, we observed both CNS and skeletal deficits in the MPS I mouse and in addition observed differences in water cross maze performance between males and females. A differential response to therapy between the sexes was also observed with respect to bodyweight gain and the water cross maze. The MPS I mouse model thus displays subtle differences in manifestation of pathology and in response to SDT of the MPS IIIA model, underscores the need to test therapies on models of specific MPS types. That SDT can be used to target CNS and skeletal pathology in MPS I, a model with no detectable residual enzyme activity, suggests that SDT may have a role to play in modulating pathology in severe MPS by delaying the onset of symptoms. However, a more thorough time course, incorporating both longer and shorter response times than analysed here, is required to confirm this concept.

## 5. Conclusions

In summary, treatment of MPS I mice with rhodamine B was able to significantly improve learning capacity in the water cross maze in female MPS I treated mice, improve grip strength in the inverted grid and have a positive effect on lumbar bone mineral volume, suggesting that SDT may be a potential therapy to improve neurological and skeletal disease in MPS I. This suggests that SDT should not be overlooked even where there is minimal or no residual enzyme activity present and may be a useful adjunct therapy option for some patients.

## Figures and Tables

**Table 1:** Brain ganglioside levels.

	Normal	MPS I Untreated	MPS I SDT
GM1	14049.98 ± 497.03	13154.62 ± 203.63	13202.02 ± 198.08
GM2	1417.10 ± 56.73	4700.51 ± 79.80 **	4607.25 ± 53.41 **
GM3	1738.95 ± 114.69	5175.89 ± 238.03 **	4890.26 ± 106.87 **

Tissue GAG levels are expressed as the average ± SEM for each group. Results are presented as pmol/g tissue. ** indicates *p* < 0.001, One-way ANOVA, Tukey’s post-hoc test, Normal vs. MPS I untreated or Normal vs. MPS I SDT.

**Table 2:** β-hexosaminidase enzyme activity levels.

	Normal	MPS I Untreated	MPS I SDT
Brain	184.26 ± 13.74	1102.85 ± 98.46 **	987.06 ± 84.54 **
Liver	1009.92 ± 103.21	9169.05 ± 443.00 **	8975.07 ± 488.27 **
Kidney	859.83 ± 0.58	5857.89 ± 11.67 **	7239.73 ± 0.001 **
Spleen	3285.37 ± 380.58	5094.60 ± 485.28 *	4412.64 ± 329.73
Heart	241.51 ± 23.76	5118.18 ± 338.74 **	4885.17 ± 414.17 **
Lung	404.69 ± 54.90	6068.17 ± 460.39 **	5755.82 ± 453.01 **

β-hexosaminidase enzyme levels are expressed as the average ± SEM for each group. Results are presented as nmol/min/mg tissue. * indicates *p* < 0.05, One-way ANOVA, Tukey’s post-hoc test, Normal vs. MPS I. ** indicates *p* < 0.001, One-way ANOVA, Tukey’s post-hoc test, Normal vs. MPS I untreated or Normal vs. MPS I SDT.

## Supplementary Materials

The following are available online, Table S1: Animal usage numbers.
