# Supplementary material for: Substrate Deprivation Therapy to Reduce Glycosaminoglycan Synthesis Improves Aspects of Neurological and Skeletal Pathology in MPS I Mice
Source: Diseases. 2017 Feb 23;5(1):5. doi: 10.3390/diseases5010005 (PMC5456338; doi:10.3390/diseases5010005)
Supplement: Supplementary file 1 [file diseases-05-00005-s001.pdf]

# Supplementary Materials: Substrate Deprivation Therapy to Reduce Glycosaminoglycan Synthesis Improves Aspects of Neurological and Skeletal Pathology in MPS I Mice

**Ainslie L. K. Derrick-Roberts, Matilda R. Jackson, Carmen E. Pyragius and Sharon Byers**

**Table S1.** Animal numbers.

Bodyweights

| | | 1 mth | 2 mth | 3 mth | 4 mth | 5 mth | 6 mth |
|---|---|---|---|---|---|---|---|
| Normal | Female | n=14 | n=13 | n=15 | n=12 | n=9 | n=10 |
| | Male | n=14 | n=14 | n=14 | n=11 | n=10 | n=10 |
| MPS I | Female | n=11 | n=11 | n=14 | n=10 | n=10 | n=6 |
| | Male | n=17 | n=16 | n=13 | n=12 | n=8 | n=6 |
| MPS I SDT | Female | n=4 | n=8 | n=8 | n=8 | n=8 | n=8 |
| | Male | n=7 | n=8 | n=8 | n=8 | n=8 | n=8 |

Biochemistry: GAG and β-hexosaminidase enzyme activity

| | | |
|---|---|---|
| Normal | Female | n=5 |
| | Male | n=7 |
| MPS I | Female | n=7 |
| | Male | n=3 |
| MPS I SDT | Female | n=6 |
| | Male | n=7 |

Behaviour

| | | IVG | OF | RR | WXM 4mth | WXM 6mth |
|---|---|---|---|---|---|---|
| Normal | Female | n=11 | n=9 | n=9 | n=3 | n=4 |
| | Male | n=10 | n=10 | n=10 | n=5 | n=4 |
| MPS I | Female | n=11 | n=10 | n=10 | n=6 | n=4 |
| | Male | n=10 | n=8 | n=8 | n=4 | n=4 |
| MPS I SDT | Female | n=8 | n=8 | n=8 | n=6 | n=2 |
| | Male | n=8 | n=8 | n=8 | n=4 | n=4 |

Bone

| | | |
|---|---|---|
| Normal | Female | n=3 |
| | Male | n=3 |
| MPS I | Female | n=6 |
| | Male | n=3 |
| MPS I SDT | Female | n=3 |
| | Male | n=3 |
